# A linear matric inequality based multi-loop PI control design for coupled multivariable liquid level system

**DOI:** 10.1038/s41598-025-89865-6

**Published:** 2025-03-14

**Authors:** Soumya Ranjan Mahapatro, Chandramauleshwar Roy, Raju Patel, Satyajit Das

**Affiliations:** 1 School of Electronic Engineering, Vellore Institute of Technology, Kelambakkam Road, Chennai, 600127 Tamil Nadu India; 2 School of Electrical Engineering, Vellore Institute of Technology, Vellore, India

**Keywords:** Decoupling Control, Process control, Linear matrix inequality, Robustness, Electrical and electronic engineering, Applied mathematics

## Abstract

This paper presents design and development of an optimal robust PI controller based on linear matrix inequalities for a typical industrial process control system. In this method, the design problem has been transformed into a state feedback controller design problem for an augmented uncertainty MIMO system. In this work, a dynamic decoupler is designed to handle loop interaction.  The control problem is then solved for the obtained decoupled subsystem by employing a constraint optimization approach, ensuring compliance with a defined LQ cost objective function. Simulation results verify the efficacy of the proposed controller, presenting its effectiveness in achieving both set-point accuracy and disturbance attenuation. Furthermore, the study employs disk margin analysis to ascertain safe ranges for gain and phase margin.

## Introduction

Many dynamic process control systems involve time delay due to the presence of feedback system and cross coupling among its control variable due to the multi input and multi output (MIMO) control structure. The presence of time delay and cross coupling may cause the inappropriate control of output variables and potentially destabilizing the control process^1–3^. This necessitates the accurate design of controller to cancel out the adverse effect of inherent time delay and cross coupling. In view of this the design of the MIMO control systems is primarily categorized into centralized control structure and decentralized control structure. The design of centralized control structure incorporate the off-diagonal controller to eliminate the interactions between control variables. This design of centralized controller is more complex due to the involvement of large no of control variable.

To overcome this issue, a decentralized control structure is developed which requires the design and tuning of only the diagonally controller element to cancel out the interaction between the control variable, and this causes the control of the MIMO system control variable similar to a single input single output (SISO) system. The decentralized control structure may be achieved with different control strategy as described in the literature^4^. In^5^ a detuning based decentralized control is proposed that interaction between coupled control loops are eliminated by dropping the off diagonal element of the plant and the diagonal element of the plant are detuned with a biggest log modulus^6,7^. PI controller is designed for the both the control loops with Ziegler Nichols method. The off diagonal elements are dropped out with a pole placement technique by shifting the dominant pole to a stable desired location^8^ else with a root trajectory method as proposed in^9^. All the detuned methods discussed in literature are having simple implementation procedure however, these detuned methods cannot eliminate the interaction completely causing an inappropriate control of the MIMO plant. In^10^, the multi loop control structure of the plant is first decomposed to equivalent independent control structure by applying equivalent open loop transfer function and internal model control based PI controller is designed for each independent control structure. A sequential loop closing method (SLCM) is designed in^11,12^ to sequentially control the inner and outer loop of a MIMO system. In SLCM the performance of the controller is highly dependent on the selection of the design sequence of design of control loop and the interaction of the coupled looped are taken in to consideration sequentially as discussed in the^13^^14^. However, the SLCM require minimal process information on the other hand require the repeated tuning sequentially for all the coupled control loop. The control algorithms of the aforesaid methods are incapable to handle the time delay arises due to the feedback loop of the MIMO system and this may causes the instability of the dynamic system. To outmatch the aforesaid issues a decoupler is designed by decoupling the MIMO system with independent multi-loop SISO system. A PID based decoupler having biased relay identification and tuning the controller gains with minimal weighed integral square error and with optimal integral absolute error and complementary sensitivity based on max algorithim are proposed in^15,16^respectively. In^17^, a decoupler is designed to minimise the interaction between the loops and further PI controller is designed based on gain margin and phase margin for diagonal dominant MIMO system to achieve robustness of the system. Further, the design of a decoupler and its controller for restructured diagonal matrix with PID based root locus technique is proposed in^18^. A modified PI controller design based on minimum-phase and non-minimum phase systems is proposed in^19^ to control the decoupled system and this enhancing the steady state performance of the MIMO system. To enhance the robustness of the MIMO system a decoupler with a controller is proposed in^20^ based on the sensitivity analysis of the measurement noise of a MIMO system. In the aforesaid discussion it can be noted that the decoupler with a controller is the most suitable design to cancel out the interaction between the variables.

The aforementioned control structure are popular due to its simple design and less computational burden. However, on the other hand, addressed approaches exhibit higher overshoot and large settling time. Moreover, the control structure in^19^^20^^17^ cannot handle parametric uncertainty and model uncertainty and hence do not guarantee robustness. In view of this, advanced decoupler based on robust controllers are proposed in literature. A decoupler with linearized state space model based predictive control for the restructured diagonal matrix of the MIMO system^21^, nonlinear back stepping based control structure in^22^and a fuzzy logic based control structure in^23^is proposed in literature. The design of the aforesaid controller can improve the transient and steady state performance of the plant. However, the design of the reported approaches are quite complex and involves with more computational burden. It needs the accurate plant parameter for the optimal performance of the controller. To overcome the challenges associated with existing methods, this work proposes a robust optimal PI controller designed using linear matrix inequality (LMI) optimization. This approach aims to achieve enhanced robustness, optimal performance, and computational efficiency in MIMO system control.

The rest of the paper is organized as follows. In section 2 modeling of multivariable liquid level system is presented. Decoupler design is described in section 3 The proposed controller design is described in section 4 Simulation results including robustness analysis are provided in section 5 Finally, conclusions are drawn in Section 6.

## Modelling of multivariable liquid level system

**Fig. 1:**
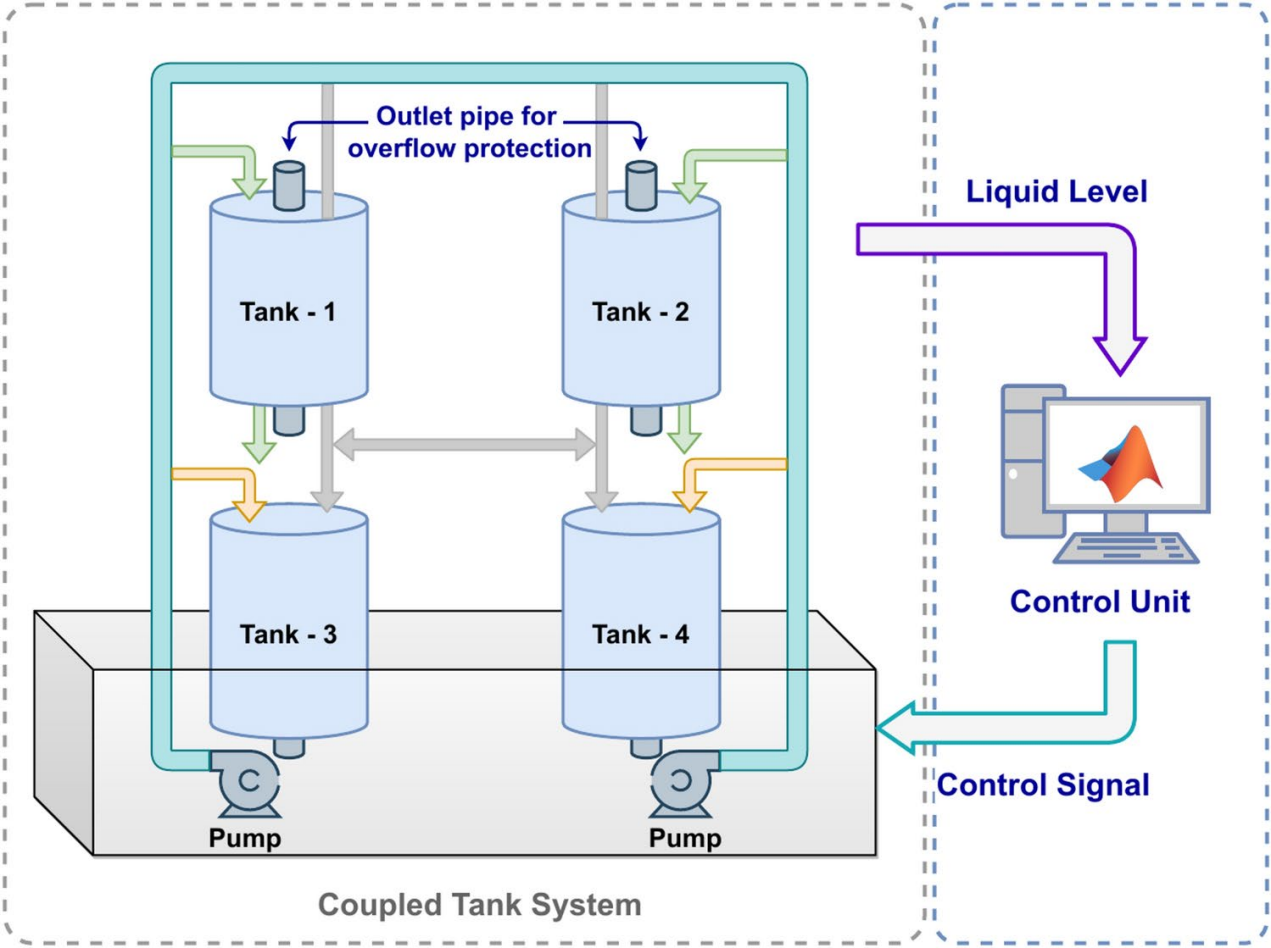
Schematic Representation of multivariable liquid level system.

In Fig. 1, the fundamental schematic design of a MIMO tank system is presented where the water level is the output and control voltage is the input. This interconnected system involves four tanks, each equipped with an output line to remove excess water from the reservoir. Significantly, the fifth tank, positioned at the bottom, functions as a reservoir for storing water. The water level in each tank is monitored by dedicated level sensors attached to the base of each tank. To facilitate water flow within the system, the reservoir tank is connected to two pumps designed to lift water from the bottom to the top. The dynamic model of this interconnected liquid level system, as detailed in^24^, is developed utilizing the mass-balance principle. This principle establishes a correlation between the water levels in the various tanks and the input applied to the pumps. The interconnected nature of the system necessitates a comprehensive understanding of its dynamic behavior to enable effective control and regulation of water levels. The dynamic model can be expressed as

1 $$\begin{aligned} \frac{d{{h}_{1}}(t)}{dt}=-\frac{{{a}_{1}}}{A}\sqrt{2g{{h}_{1}}(t)}+\eta {{u}_{1}}(t)-\frac{{{a}_{12}}}{A}\sqrt{2g({{h}_{1}}(t)-{{h}_{2}}(t))} \end{aligned}$$

2 $$\begin{aligned} \frac{d{{h}_{2}}(t)}{dt}=-\frac{{{a}_{2}}}{A}\sqrt{2g{{h}_{2}}(t)}+\eta {{u}_{2}}(t)-\frac{{{a}_{12}}}{A}\sqrt{2g({{h}_{1}}(t)-{{h}_{2}}(t))} \end{aligned}$$

Employing taylor series expansion on Equations (1) - (2) one obtains a linear model as

$$\begin{aligned}&\Delta {{H}_{1}}(s)={{f}_{1}}\Delta {{U}_{1}}(s)+{{f}_{2}}\Delta {{U}_{2}}(s), \\&\Delta {{H}_{2}}(s)={{f}_{1}}\Delta {{U}_{2}}(s)+{{f}_{2}}\Delta {{U}_{1}}(s) \end{aligned}$$

where

$$\begin{aligned} {{f}_{1}}(s)= & \frac{\eta .\left[ s+\left( {{k}_{2}}+{{k}_{12}} \right) \right] }{\left[ s+\left( {{k}_{1}}+{{k}_{12}} \right) \right] .\left[ s+\left( {{k}_{2}}+{{k}_{12}} \right) \right] -{{k}^{2}}_{12}}, \\ {{f}_{2}}(s)= & \frac{\eta .{{k}_{1}}_{2}}{\left[ s+\left( {{k}_{1}}+{{k}_{12}} \right) \right] .\left[ s+\left( {{k}_{2}}+{{k}_{12}} \right) \right] -{{k}^{2}}_{12}} \end{aligned}$$

## Design of decoupler

**Fig. 2:**
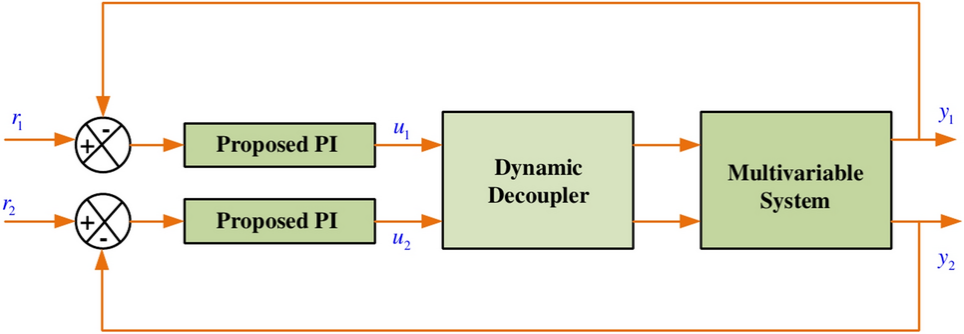
Schematic Representation of TITO control structure.

To compensate the loop interactions in a multivariable system, the decouplers are often employed. In general, a decoupler network is integrated into a multivariable process; it first eliminates the loop interaction from the system. Then, the process can be treated as multiple single loop subsystems. Compared to all dynamic decoupling approach, the inverted decoupling approach is one of the most significant dynamic decoupling methods rather than other two i.e. simplified as well as ideal decoupling scheme. Alternatively, this decoupling structure is named feed-forward decoupling control. Figure 2, depicts the most generalized inverted decoupling structure for a TITO process. In the above Fig. 2, $r_1$, $r_2$ are the reference variable, $u_1$, $u_2$ are the input from plant to the diagonal elements, and $y_1$, $y_2$ are the plant outut respectively. For inverted decoupling, the decoupler matrix is derived as

3 $$\begin{aligned} \left[ \begin{matrix} {{u}_{1}} \\ {{u}_{2}} \\ \end{matrix} \right]= & \left[ \begin{matrix} 1 & 0 \\ 0 & 1 \\ \end{matrix} \right] \left[ \begin{matrix} {{c}_{1}} \\ {{c}_{2}} \\ \end{matrix} \right] +\left[ \begin{matrix} 0 & -\frac{{{G}_{12}}}{{{G}_{11}}} \\ -\frac{{{G}_{21}}}{{{G}_{22}}} & 0 \\ \end{matrix} \right] \left[ \begin{matrix} {{u}_{1}} \\ {{u}_{2}} \\ \end{matrix} \right] \nonumber \\ D= & {{\left[ \begin{matrix} 1 & -\frac{{{G}_{12}}}{{{G}_{11}}} \\ -\frac{{{G}_{21}}}{{{G}_{22}}} & 1 \\ \end{matrix} \right] }^{-1}} \end{aligned}$$

The application of inverted decoupling to some processes is constrained by some of the critical parameters that determine the decoupler design. The constraint is as follows:

- $-\frac{{{G}_{12}}(s)}{{{G}_{11}}(s)}$ and $-\frac{{{G}_{21}}(s)}{{{G}_{22}}(s)}$ must be strictly proper or proper function
- ${{G}_{12}}(s)$ delay must be greater than or equal to ${{G}_{11}}(s)$ and ${{G}_{12}}(s)$ order must be greater than or equal to ${{G}_{11}}(s)$
- ${{G}_{21}}(s)$ delay must be greater than or equal to ${{G}_{22}}(s)$ and ${{G}_{21}}(s)$ order must be greater than or equal to ${{G}_{22}}(s)$

If any plant transfer function matrix satisfies these above constraints, then only the inverted decoupling strategy can be used to remove the coupling impact from the system.

## Proposed LMI inequality multiloop PI controller

Classical design based PID controller are widely used in process control industries due to their simple structure. However, in a multi-input and multi-output system, the PID controller gains are designed by considering a single-input and single-output system (SISO). In this work a robust LMI-based PI controller is synthesized to control the MIMO coupled tank system. The design of the control structure is first converted to a state feedback-based LMI and then solved with the convex optimization technique. The proportional gain for the inner and outer control loop is designed with the static feedback gain K to obtain the fastest response to the error.

Typically, the additional variables are selected as the integral of the required output to achieve zero steady-state error. If an auxiliary state τ is introduced as:

4 $$\tau =-\int {\left( r-{{x} _ {1}} \right) }dt$$,

where r represents the desired trajectory of the output, the PI-like state feedback control problem can be formulated as follows.

Consider an LTI system with disturbance *w* given by:

5 $$\begin{aligned} \dot{x}=  Gx+Hu+{{H}_{w}}w \end{aligned}$$

6 $$\begin{aligned} y= Jx \end{aligned}$$

Consider an objective function for Eq. (4) as:

7 $$\begin{aligned} J=\int \limits _{0}^{\infty }{\left( {{x}^{T}}(t)Mx(t)+{{u}^{T}}(t)Nu(t) \right) }dt \end{aligned}$$

where *M* and *N* are semidefinite positive symmetric matrices. The control law is given by:

8 $$\begin{aligned} u=-Kx=-{{N}^{-1}}{{H}^{T}}S \end{aligned}$$

In Eq. (6), *S* denotes a positive definite matrix, which can be obtained as the solution of the Algebraic Riccati Equation (ARE). It is well known that the system input-output gain for finite energy can be measured by the $H\infty$ norm of a transfer function. This constraint is beneficial for achieving good performance in the case of disturbance and uncertainty, ensuring robust stability.

9 $$\begin{aligned} {{\left\| {{T}_{zw}} \right\| }_{\infty }}\le \sigma \ \end{aligned}$$

The inequality in Eq. (7) can be recast as an LMI using the Schur complement as follows:

10 $$\begin{aligned} \left[ \begin{matrix} G\hat{S}+HY+\hat{S}{{G}^{T}}+{{Y}^{T}}{{H}^{T}} & \hat{S}{{H}_{w}} & {{J}^{T}} \\ H_{w}^{T}\hat{S} & -\sigma I & 0 \\ J & 0 & -\sigma I \\ \end{matrix} \right] \le 0 \end{aligned}$$

### Proof of Lemma-2

On substituting the control law u = kx, in Eq. (4), the closed system dynamics are:

11 $$\begin{aligned} \dot{x}= & Gx+HKx+{{H}_{w}}w = & {{G}_{c}}x+{{H}_{w}}w \end{aligned}$$

where ${{G}_{c}}=(G+HK)x$. Now, considering Eqs. (8), (9) the system can be rewritten as:

12 $$\begin{aligned} \left[ \begin{matrix} G_{c}^{T}S+S{{G}_{c}} & S{{H}_{w}} & {{J}^{T}} \\ H_{w}^{T}S & -\sigma I & 0 \\ J & 0 & -\sigma I \\ \end{matrix} \right] \le 0 \end{aligned}$$

13 $$\begin{aligned} G_{c}^{T}S+S{{G}_{c}}+S{{H}_{w}}{{\left( \sigma i \right) }^{-1}}H_{w}^{T}S+{{J}^{T}}{{(\sigma i)}^{-1}}J<0 \end{aligned}$$

Employing ${{G}_{c}}=(G+HK)$ in Eq. (10), we obtain:

14 $$\begin{aligned} {{K}^{T}}{{H}^{T}}S+{{G}^{T}}S+SG+SHK+S\Gamma S +\gamma <0 \end{aligned}$$

By pre and post multiplying ${{S}^{-1}}$ on Eq. (11), we get:

15 $$\begin{aligned} {{S}^{-1}}{{G}^{T}}S{{S}^{-1}}+{{S}^{-1}}SG{{S}^{-1}}+{{S}^{-1}}{{K}^{T}}{{H}^{T}}S{{S}^{-1}}\nonumber \\ +{{S}^{-1}}SHK{{S}^{-1}}+{{S}^{-1}}S\Gamma S{{S}^{-1}}+{{S}^{-1}}\gamma {{S}^{-1}}<0 \end{aligned}$$

where:

16 $$\begin{aligned} \Gamma= & {{H}_{w}}{{(\sigma i)}^{-1}}H_{w}^{T}\end{aligned}$$

17 $$\begin{aligned} \gamma= & {{J}^{T}}{{(\sigma i)}^{-1}}J \end{aligned}$$

Setting $y=K\hat{S}$ and $\hat{S}={{S}^{-1}}$, we arrive at:

18 $$\begin{aligned} \hat{S}{{G}^{T}}+G\hat{S}+\hat{S}{{K}^{T}}{{H}^{T}}+HK\hat{S}+\Gamma +\hat{S}\gamma \hat{S}<0 \end{aligned}$$

### Note 1: Controller Parameter Design Steps

- Identify the state space model for the process.
- Determine the LQR weighting matrix parameter values *Q* and *R*. The transient response of the dynamic system is influenced by the location of the poles. The constraints for the placement of the closed-loop poles can be found by choosing a suitable LMI region.
- Evaluate the feasible solution for ${{\hat{S}}^*}{Y}$ for the defined LMIs in Eq. (13).
- Compute the optimal controller parameter values by solving the matrix equation:
  19 $$\begin{aligned} K=-{{Y}^{*}}{{(S)}^{-1}} \end{aligned}$$
- Substitute the obtained values into the controller structure to achieve the desired system response.

### Remark 1: LMI region

**Fig. 3:**
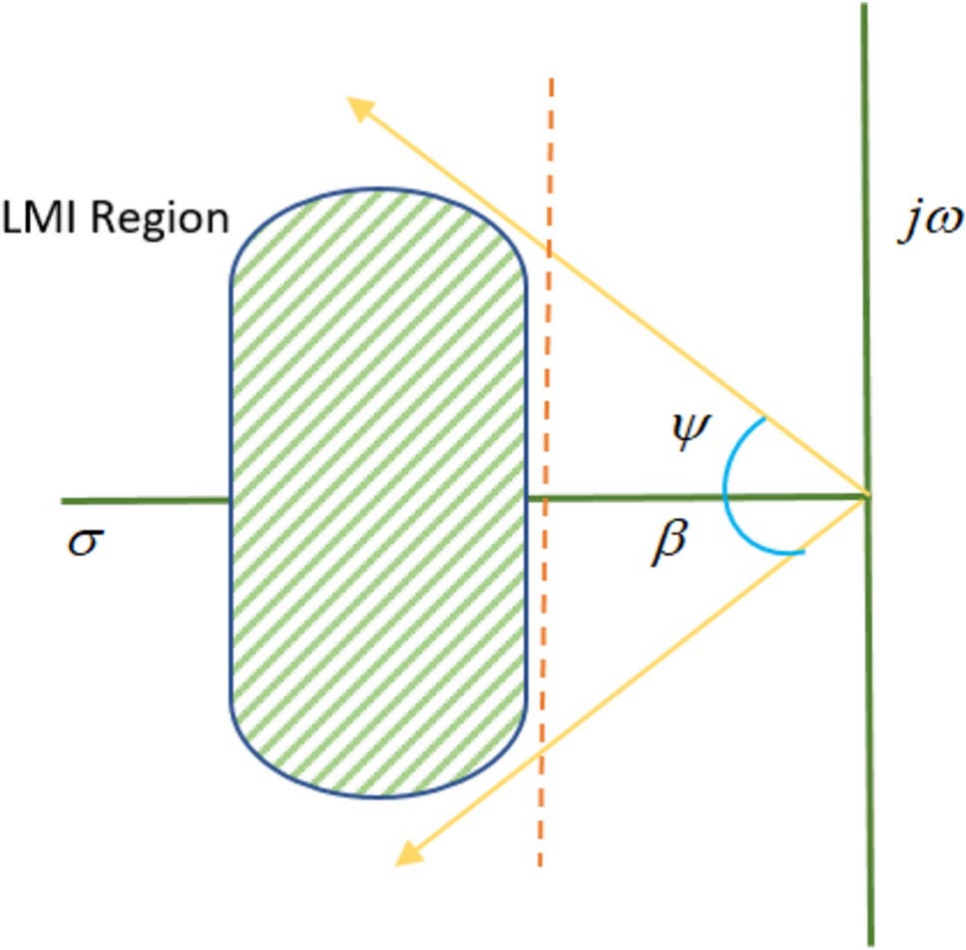
Generalized representation pole placement LMI region.

A subset $\textrm{Z}$ of the complex plane is defined as the LMI region in Fig. 3, if there exist a symmetric matrix $M = {M^T} = {\left[ {{\alpha _{ij}}} \right] _{1 \le i,j \le p1}}$ and $N = {\left[ {{\beta _{ij}}} \right] _{_{1 \le i,j \le p1}}}$ such that $\textrm{Z} = \left\{ {\textrm{S} \in \textrm{H}:M + SN + {N}{S^T} < 0} \right\}$ if and only if there exists a symmetric positive matrix ${\hat{S}}$ satisfying the given LMIs, which can be obtained by substituting the value of M, S, N and ${\bar{N}}$ in the following equation:

20 $${\left[ {{\alpha _{ij}}S + {\beta _{ij}}S + {\beta _{ij}}{S^T}} \right] _{1 \le i,j \le p1}}$$

The condition for stability is given by:

21 $$\begin{aligned} \hat{S}{{G}^{T}}+G\hat{S}+{{Y}^{T}}{{H}^{T}}+HY+\Gamma +\hat{S}\gamma \hat{S}\le 0 \end{aligned}$$

22 $$\begin{aligned} \left[ \begin{matrix} G\hat{S}+HY+\hat{S}{{G}^{T}}+{{Y}^{T}}{{H}^{T}} & \hat{S}{{H}_{w}} & {{J}^{T}} \\ H_{w}^{T}\hat{S} & -\sigma I & 0 \\ J & 0 & -\sigma I \\ \end{matrix} \right] \le 0 \end{aligned}$$

The control gain *K* is computed as:

23 $$\begin{aligned} K=-{{Y}^{*}}{{(S)}^{-1}} \end{aligned}$$

On solving the above equations, the obtained parameter values of *K* are as follows:

24 $$\begin{aligned} \begin{array}{l} {K_1} = \left[ {\begin{array}{*{20}{c}} {{K_{{P_1}}}}& {{K_{{I_1}}}} \end{array}} \right] = [2.4242\,\,\,0.05]\ \end{array} \end{aligned}$$

25 $${K_2} = \left[ {\begin{array}{*{20}{c}} {{K_{{P_2}}}}& {{K_{{I_2}}}} \end{array}} \right] = [3.8244\,\,\,0.01]$$

## Results and discussions

This section describes the presentation of simulation results to demonstrate the appropriateness and performance of the proposed controller. In this work, a coupled tank multi-variable liquid level system has been considered as a case study to validate the proposed method. After substituting the parameters of the coupled-tank^24^, the linearized plant model is obtained as:

26 $$\begin{aligned} G=\left[ \begin{array}{ll} \frac{0.0822s+0.0025729}{{{s}^{2}}+0.062s+0.00044561} & \frac{0.00186594}{{{s}^{2}}+0.062s+0.00044561}\\ \\ \frac{0.0135s+0.000414}{{{s}^{2}}+0.062s+0.00044561} & \frac{0.000307}{{{s}^{2}}+0.062s+0.00044561} \end{array} \right] \end{aligned}$$

By utilizing Eq. (3), the resulting inverted decoupling controller matrix for the obtained model Eq. (26) of the coupled tank system is as

27 $$\begin{aligned} D={{\left[ \begin{matrix} 1 & \frac{0.00186594}{0.0822s+0.0025729} \\ \frac{0.013s+0.000414}{0.000307} & 1 \\ \end{matrix} \right] }^{-1}} \end{aligned}$$

**Fig. 4:**
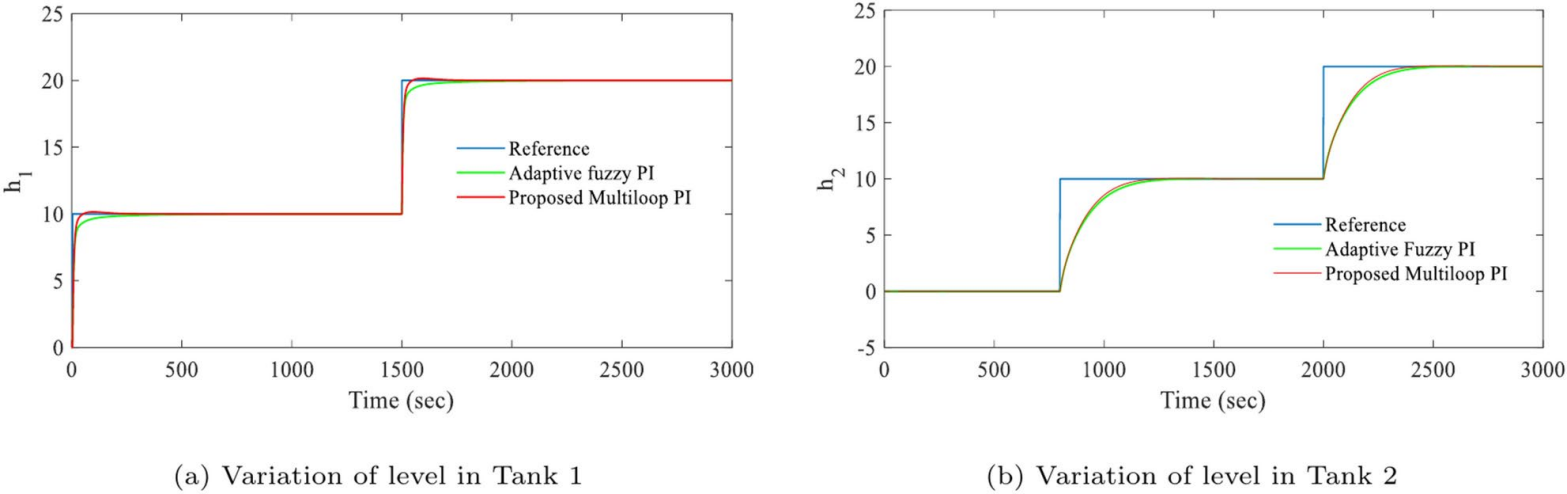
Simulation results of set-point tracking for nominal system.

**Fig. 5:**
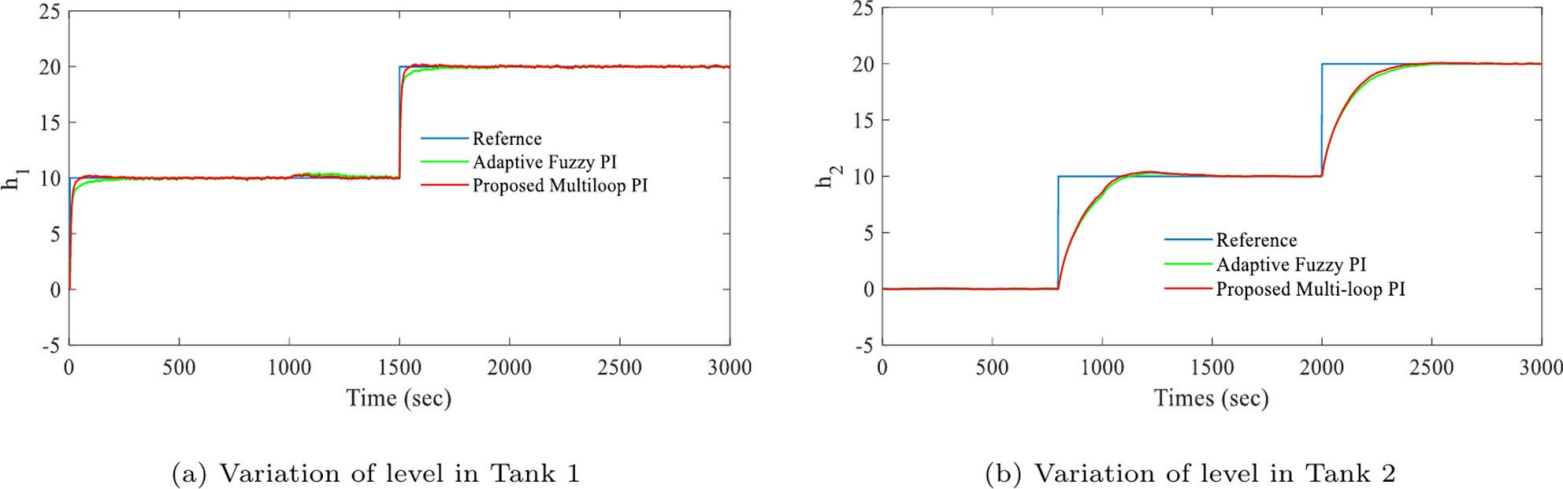
Simulation results of set-point tracking for nominal system with stochastic noise.

To validate the efficacy of the controller two scenario has been considered namely (a)nominal system (b) nominal system with stochastic noise and load perturbation. The effectiveness of the proposed LMI-based multi-loop PI controller is demonstrated by comparing its responses with those of an adaptive fuzzy PID controller. The corresponding simulation results are shown in Figs. 4 and 5. The obtained simulation response indicates that the desired levels are attained in both tanks without affecting the other level.

**Fig. 6:**
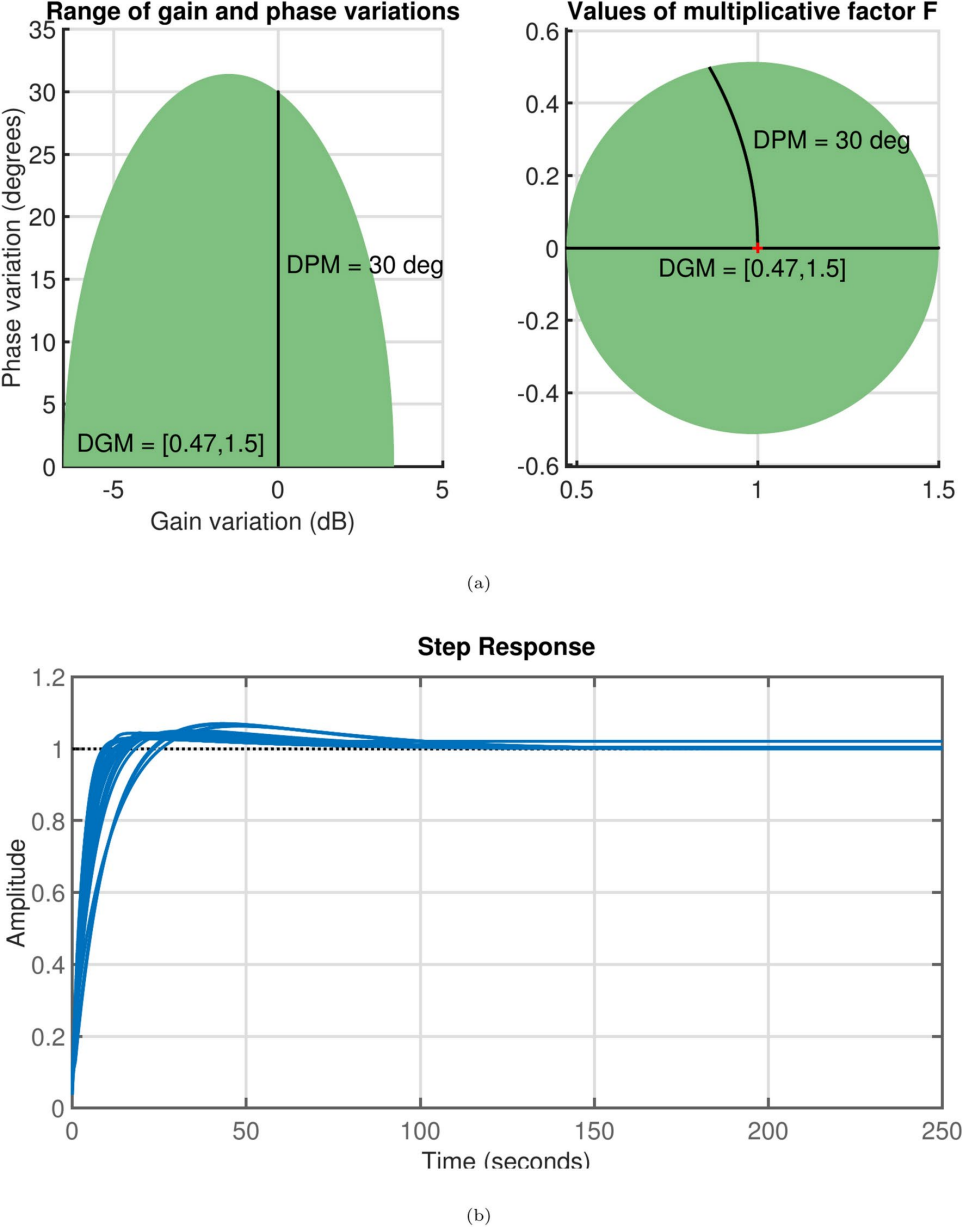
(**a**) Gain, phase change before adding uncertainty for $\sigma$=*Tight*, (**b**) Step Response.

**Fig. 7:**
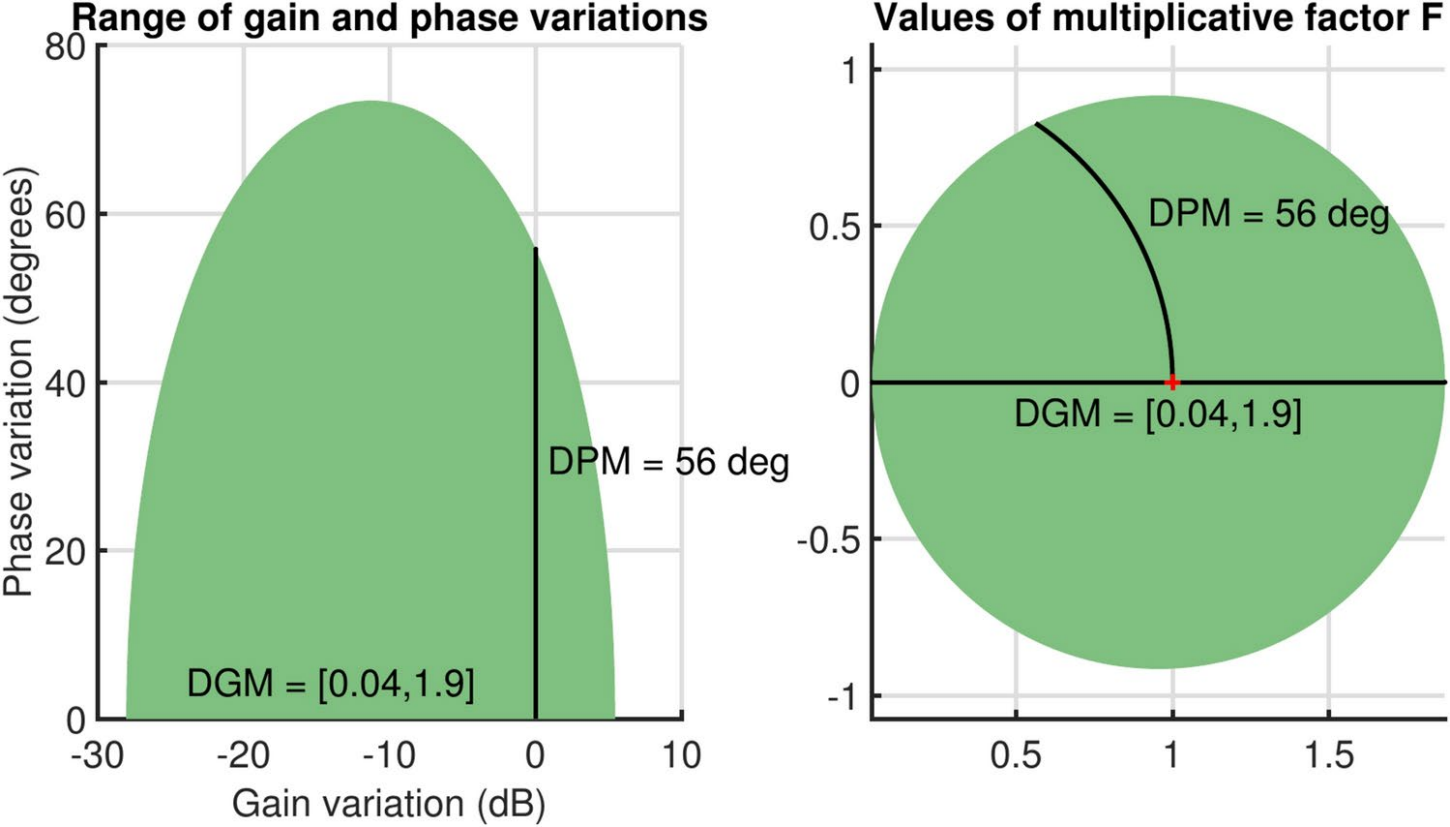
Safe Ranges of gain and phase margin.

**Fig. 8:**
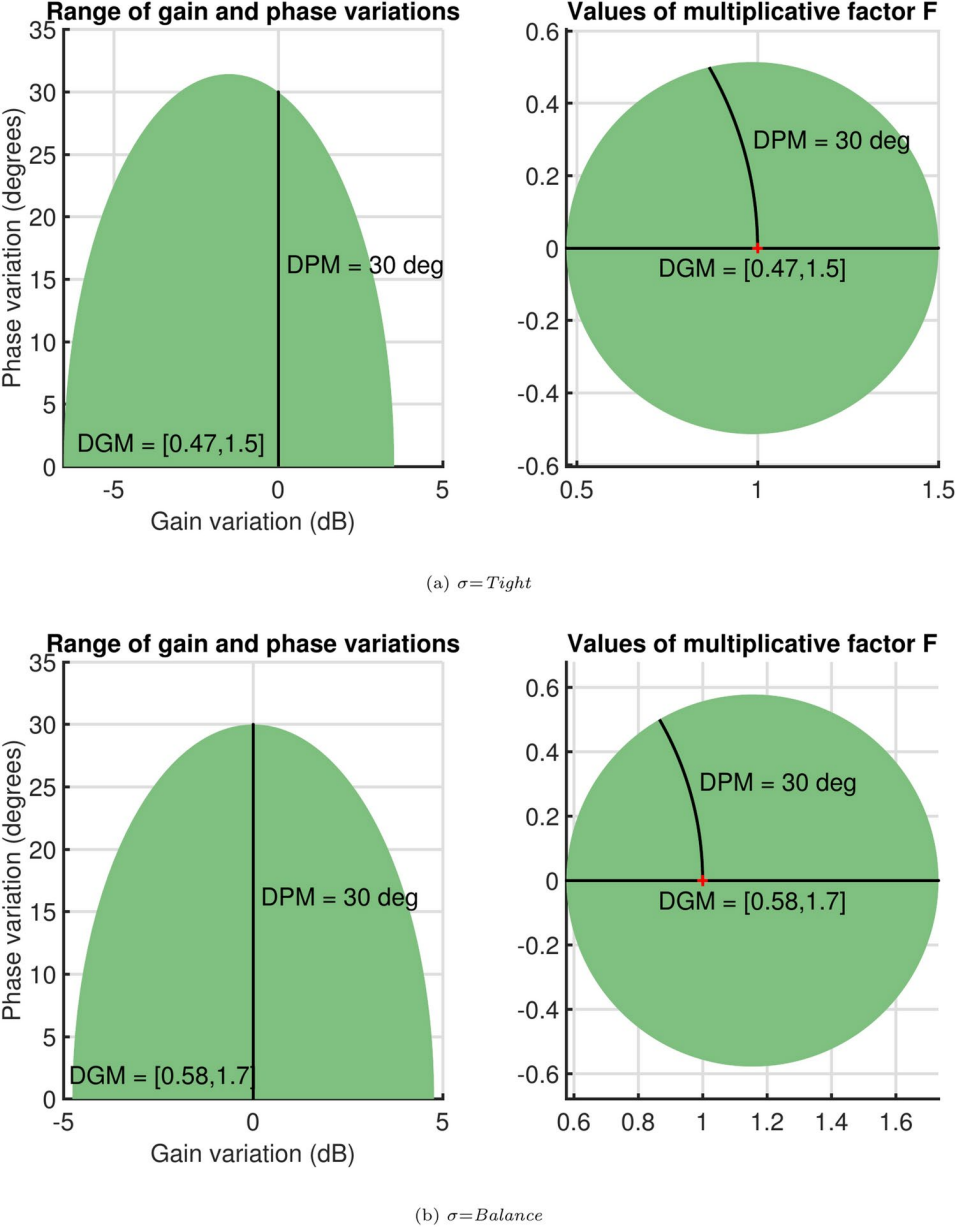
Gain, phase change after adding uncertainty.

The open-loop gain is assumed to vary by ±50% for stability evaluation, while the phase fluctuates by $\pm 30^o$. Setting $\sigma$ = 1 (nominal value) ensures that the smallest disk captures the specified gain and phase uncertainties. The least uncertainty disk in Fig. 6a shows that a phase shift of $\pm 30^o$ is possible when the relative gain fluctuation is limited to the range [0.47, 1.5]. This indicates that although the phase may vary by $\pm 30^o$ without altering gain, the system can endure a 47$\%$ reduction in gain without any phase variation. Amid these uncertainties, Fig. 6b illustrates the closed-loop reaction, demonstrating that the system remains stable despite a $\pm 30^o$ phase shift and a 150% gain augmentation. However, the system is less robust. Hence, the robust stability margin is determined as 1.7 which infers that the system can tolerate uncertainty up to 1.7 times the specified value. Figure 7 presents the safe ranges for gain and phase with uncertainties. The relative gain change will be between [0.04,1.9] with a phase change of $\pm 56^o$. Further, the smallest disc that captures the specified gain and phase uncertainty is presented in Fig. 8a. The range is given by [0.47,1.5]. Further, when $\sigma$ is *balance* equal amounts of increment and decrement in relative gain can be modeled as shown in Fig. 8b. The variation is between [0.58,1.7].

**Fig. 9:**
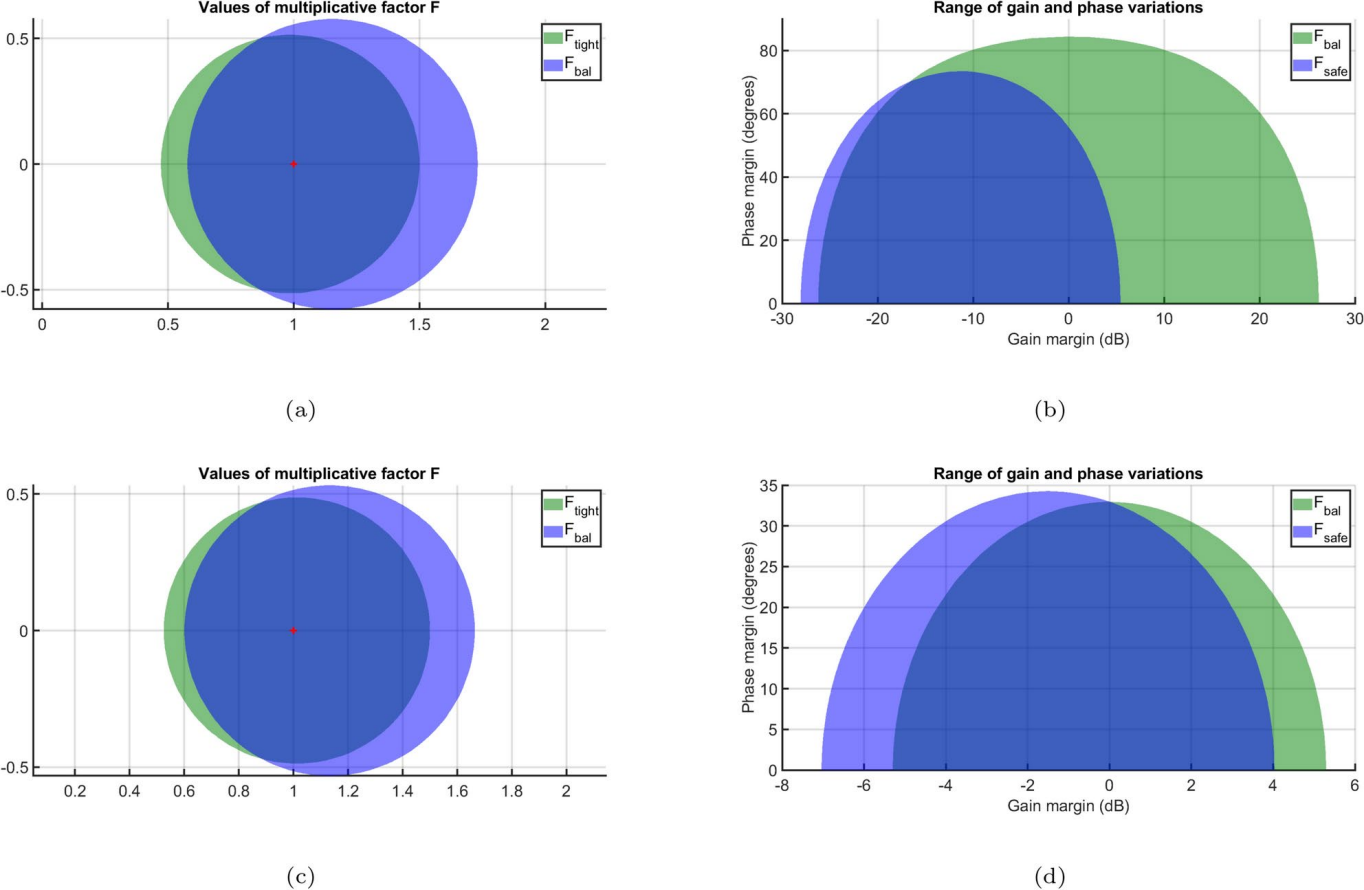
Safe ranges for gain and phase.

However, when both gain and phase vary, the variations will remain inside the shaded region on the left side. A comparative analysis for the two modelswith uncertainty values set as $\sigma$ tight and balanced for a 50$\%$ gain variation and $\pm 30^o$ phase variation is presented in Fig. 9a. Here, the parameter $\sigma$ corresponds to the balanced disc. It is observed that the uncertainty disc is larger for the balanced case. Furthermore, Fig. 9b illustrates the safe ranges of gain and phase margin for both models. Similarly, Fig. 9c and d present the comparative analysis and safe ranges for the other diagonal element, considering a 50$\%$ open-loop gain variation and a $\pm 28^o$ phase variation.

## Conclusion

In this paper, an LMI based optimal decentralized PI controller is proposed for Two-Input Two-Output liquid level benchmark process to meet both robustness and loop performance requirements simultaneously. This study presents an LMI-based approach for directly synthesizing PID controllers that achieve both LQR and h infinity performance. Furthermore, this research outlines a strategy for designing MIMO PID controllers using optimal LMI-LQR techniques. Table 1 presents the performance evaluation of designed controller, while robustness is analyzed under model uncertainty and external disturbances. The simulation results validate the effectiveness of the proposed controller by demonstrating its capability to attain precise set points and effectively suppress disturbances. In addition, disk margin analysis is utilized in the study to determine acceptable ranges for phase margin and gain. The obtained results reveals that LMI based decentralized PI controller provides adequate performance in all considered unavoidable realtime scenarios.

**Table 1:** Performance assesment.

Controller	Input (u) - Output (y)	${{M}_{s}}$	${{\left\| u \right\| }_{\infty }}$	${{e}_{t}}$	IAE	TV
LMI Based Optimal Robust Decentralized PI	${{u}_{1}}-\,{{y}_{1}}$	1.04	132.68	0.58	452.2	9.28
	${{u}_{2}}-\,{{y}_{2}}$	1.01	166.5	1.92	642.5	8.89
Decentralized PI	${{u}_{1}}-\,{{y}_{1}}$	1.31	145.75	0.78	511	10.14
	${{u}_{2}}-\,{{y}_{2}}$	1.22	163.32	2.56	760.3	9.27

## Data Availability

Any data that support the findings of this study are included within the article.

## References

[1] Espitia, H., Machón, I. & López, H. Proposal of a Compact Neuro-Fuzzy Adaptive Controller for Filling Regulation of Two Coupled Spherical Tanks. Int. J. Fuzzy Syst. 1–19 (2024).

[2] El-Nagar, A.M. & Abdo, M.I. Development of sliding mode control based on diagonal recurrent neural network for coupled tank system. Neural Comput. Appl. 1–15 (2024).

[3] Wang, J., Wu, Y., Chen, C.P., Liu, Z. & Wu, W. Adaptive PI event-triggered control for MIMO nonlinear systems with input delay. Inf. Sci. 120817 (2024).

[4] Nagarajapandian, M., Kanthalakshmi, S., Devan, P. A. M. & Bingi, K. Optimal iterative learning PI controller for SISO and MIMO processes with machine learning validation for performance prediction. *Sci. Rep.* **14**(1), 23568 (2024). 39384628 10.1038/s41598-024-74813-7 PMC11464494

[5] Ziegler, J. G. & Nichols, N. B. Optimum settings for automatic controllers. *Trans. Am. Soc. Mech. Eng.* **64**(8), 759–765 (1942).

[6] Luyben, W. L. Simple method for tuning siso controllers in multivariable systems. *Ind. Eng. Chem. Process Des. Dev.* **25**(3), 654–660 (1986).

[7] Chien, I.-L., Huang, H.-P. & Yang, J.-C. A simple multiloop tuning method for pid controllers with no proportional kick. *Ind. Eng. Chem. Res.* **38**(4), 1456–1468 (1999).

[8] Lee, J. & Edgar, T. F. Multiloop pi/pid control system improvement via adjusting the dominant pole or the peak amplitude ratio. *Chem. Eng. Sci.* **61**(5), 1658–1666 (2006).

[9] Zhang, Y., Wang, Q.-G. & Astrom, K. Dominant pole placement for multi-loop control systems. *Automatica* **38**(7), 1213–1220 (2002).

[10] Vu, T. N. L. & Lee, M. Independent design of multi-loop pi/pid controllers for interacting multivariable processes. *J. Process Control* **20**(8), 922–933 (2010).

[11] Shiu, S.-J. & Hwang, S.-H. Sequential design method for multivariable decoupling and multiloop pid controllers. *Ind. Eng. Chem. Res.* **37**(1), 107–119 (1998).

[12] Hovd, M. & Skogestad, S. Sequential design of decentralized controllers. *Automatica* **30**(10), 1601–1607 (1994).

[13] Xiong, Q. & Cai, W.-J. Effective transfer function method for decentralized control system design of multi-input multi-output processes. *J. Process Control* **16**(8), 773–784 (2006).

[14] Lakshmanaprabu, S., Elhoseny, M. & Shankar, K. Optimal tuning of decentralized fractional order pid controllers for tito process using equivalent transfer function. *Cogn. Syst. Res.* **58**, 292–303 (2019).

[15] Jevtović, B. T. & Mataušek, M. R. Pid controller design of tito system based on ideal decoupler. *J. Process Control* **20**(7), 869–876 (2010).

[16] Liu, L. et al. A review of industrial mimo decoupling control. *Int. J. Control Autom. Syst.* **17**(5), 1246–1254 (2019).

[17] Maghade, D. & Patre, B. Decentralized pi/pid controllers based on gain and phase margin specifications for tito processes. *ISA Trans.* **51**(4), 550–558 (2012). 22445395 10.1016/j.isatra.2012.02.006

[18] Wutthithanyawat, C. & Wangnipparnto, S. Design of decentralized pid controller with the root locus method based on inverted decoupling for a tito system. Interdiscip. Res. Rev. **13**(2) (2018).

[19] Begum, K. G., Rao, A. S. & Radhakrishnan, T. Enhanced imc based pid controller design for non-minimum phase (nmp) integrating processes with time delays. *ISA Trans.* **68**, 223–234 (2017). 28325526 10.1016/j.isatra.2017.03.005

[20] Abdul-Adheem, W. R., Ibraheem, I. K., Azar, A. T. & Humaidi, A. J. Improved active disturbance rejection-based decentralized control for mimo nonlinear systems: comparison with the decoupled control scheme. *Appl. Sci.* **10**(7), 2515 (2020).

[21] Guo, X., Shirkhani, M. & Ahmed, E. M. Machine-learning-based improved smith predictive control for mimo processes. *Mathematics* **10**(19), 3696 (2022).

[22] Tong, S.-C., Li, Y.-M., Feng, G. & Li, T.-S. Observer-based adaptive fuzzy backstepping dynamic surface control for a class of mimo nonlinear systems. *IEEE Trans. Syst. Man Cybern. B (Cybern.)* **41**(4), 1124–1135 (2011). 21317084 10.1109/TSMCB.2011.2108283

[23] Patel, H. R. & Shah, V. A. Decentralized stable and robust fault-tolerant pi plus fuzzy control of mimo systems: a quadruple tank case study. *Int. J. Smart Sens. Intell. Syst.* **12**(1), 1–20 (2019).

[24] Feedback Instruments Ltd: Coupled Tank System Control Experiment Manual. UK. Feedback Instruments Ltd. (2000).

